# Evaluation of Treatment in the Smart Home IRIS in terms of Functional Independence and Occupational Performance and Satisfaction

**DOI:** 10.1155/2013/926858

**Published:** 2013-11-24

**Authors:** Julija Ocepek, Anne E. K. Roberts, Gaj Vidmar

**Affiliations:** ^1^University Rehabilitation Institute, Republic of Slovenia, Linhartova 51, SI-1000 Ljubljana, Slovenia; ^2^Faculty of Health, Education and Society, University of Plymouth, SF18, Peninsula Allied Health Centre, College of St Mark & St John, Plymouth, Devon PL6 8BH, UK

## Abstract

The development of assistive technologies, home modifications, and smart homes has rapidly advanced in the last two decades. Health professionals have recognised the benefits of these technologies in improving individual's quality of life. The Smart Home IRIS was established in 2008 within the University Rehabilitation Institute in Ljubljana with the aim to enable persons with disabilities and elderly people to test various assistive technologies and technical solutions for their independent living. We investigated the effect of treatments in the Smart Home IRIS. A convenience sample of 59 persons with disabilities and elderly people (aged 24–81 years) who were treated in the Smart Home IRIS from April to December 2011 participated. Standardised instruments—the Canadian Occupational Performance Measure (COPM) and the Functional Independence Measure (FIM)—were administered at the first assessment in the Smart Home IRIS and at a second assessment at the participant's home after 6–12 months. All the outcomes statistically significantly improved from the first to the second assessment. The treatments in the Smart Home IRIS appeared to contribute to higher occupational performance and satisfaction with performance and higher functional independence of persons with disabilities and elderly people.

## 1. Introduction

In the recent years, there has been an extended development and increased prescription of assistive technologies (ATs), including smart home technologies, that help persons with disabilities and elderly to live more independently. In Slovenia, there are several barriers that thwart the implementation of ATs, such as a lack of strong political initiatives towards AT development, the cost of ATs, and no possibility to test various ATs before buying them. Provision of ATs within the Slovenian health care system differs from many European countries in the sense that persons with disabilities and elderly must buy the majority of ATs.

**Figure 1:**
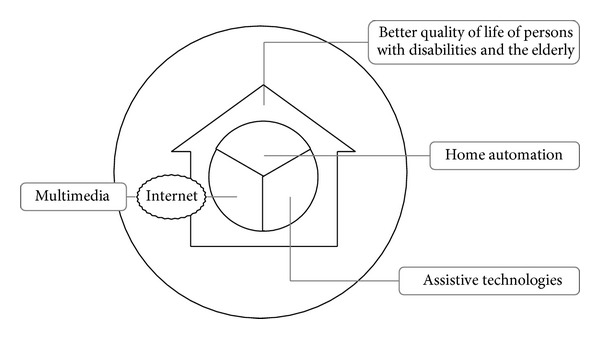
The concept of the Smart Home IRIS.

One of the solutions for enabling persons with disabilities and elderly people to receive adequate information about ATs and to test appropriate ATs was the establishment of the Smart Home Independent Residing enabled by Intelligent Solutions (IRIS), in 2008. The main aim of the Smart Home IRIS is demonstration, testing, and application of contemporary technological solutions that compensate for diverse kinds of disabilities and thereby improve the quality of life of persons with disabilities [1]. The Smart Home IRIS is a demonstration apartment at the University Rehabilitation Institute in Ljubljana, founded on the basis of numerous European smart home projects. Its concept is presented in Figure 1.

The apartment is fitted with various assistive technologies, from simple to the most advanced, which assist persons with different disabilities as well as the elderly. The Smart Home IRIS enables persons with disabilities and the elderly to achieve the highest possible level of functional independence. Adapted equipment, technical aids, and numerous contemporary electronic systems which enable a user to control his or her living environment (open the doors and windows, pull the curtains, control the TV, radio, telephone, switch on/off the heating system, etc.) in various manners (using switches, remote control, voice control, wheelchair joystick, eye control, ambient intelligence, etc.). The underlying rationale is our belief, based on our clinical experience, that controlling the living environment leads to a better quality of life.

The Smart Home IRIS is equipped with state-of-the-art information and communication technology, which is adapted to different levels and types of disability. This enables the users to communicate with the outside world, to receive remote care and remote monitoring of their health condition, and to partake in studying, work, leisure, and entertainment by means of electronic media. Individual treatments are client-centred—led by the problems identified by patients in the Canadian Occupational Performance Measure (COPM) [2] and performed by a multidisciplinary team (composed of a physiatrist, an occupational therapist, an engineer, and other professionals). The service is funded by the national health insurance company and incorporates several subservices.

Because five years have passed since the establishment of the Smart Home IRIS, evidence of its role and its contribution to the Slovenian health care system in general, and rehabilitation medicine in particular, is needed. Feedback from persons who have been treated in the Smart Home IRIS is also important for further planning of treatments, for initiating some changes of the daily practice, or for including additional services. A small initial study—a mail survey in which 117 persons with disabilities and elderly participated—was carried out during 2008 and 2009 [3]. The results provided important feedback about adequacy of treatments in the Smart Home IRIS and about satisfaction with the treatment from the users' point of view. However, due to the limitations of the research design, the acquired evidence was not strong. Therefore, the present study was conducted.

## 2. Materials and Methods

### 2.1. Study Design and Instruments

A quantitative quasi-experimental study was conducted, employing repeated measurement at two time-points without a control group. The dependent variables were the functional independence scores (total, motor, and cognitive scores on the Functional Independence Measure—FIM [4]) and the COPM performance and satisfaction ratings at the first treatment in the Smart Home IRIS and at the second assessment. The independent variables were the participants' characteristics: gender, diagnosis, age, and number of ATs used.

Three research hypotheses were tested. (1) The use of ATs and home modification has positive impact on functional independence of the persons treated in the Smart Home IRIS. (2) The use of ATs and home modification has positive impact on occupational performance and satisfaction with occupational performance of the persons treated in the Smart Home IRIS. (3) There are differences in progress with regard to the diagnosis of the persons who were treated in Smart Home IRIS and the number of assistive technologies that they used.

The COPM was used because the treatments in the Smart Home IRIS are client-centred in nature. It is a standardised individualized measure in the form of a semistructured interview designed to measure a client's self-perception of occupational performance. It also assists in setting the goals and can serve as an outcome measure to determine the degree of change in occupational performance over time as a result of intervention [5]. The focus of the COPM is on occupational performance areas, namely self-care, productivity, and leisure. The client's perspective is sought through the interview, and occupational performance problems are defined by the client. After the interview, the client uses a 0–10 scale to rate his or her perceived performance on each of the identified tasks and to rate his or her satisfaction with his or her own performance. The COPM has been reported to be a valid, reliable, clinically useful, and responsive outcome measure for occupational therapists [6]. Studies have reported the use of the COPM with a wide variety of clients in numerous different settings, including those with physical and mental health issues, all age groups, as well as clients in hospital, outpatient, and community settings [7–9]. Several studies have examined the test-retest reliability of the COPM [10–12] and the results indicate that the COPM is highly reliable (the reliability coefficients consistently exceed 0.80). The COPM has also demonstrated acceptable content, criterion, and construct validity [13–15]. Because the reproducibility of the mean performance and satisfaction scores on the COPM has been found to be moderate but poor for the scores of the separate problems [16], the mean scores of performance and satisfaction were used.

The FIM is the most widely accepted functional assessment measure used with all diagnostic groups within the adult rehabilitation population, as evidenced in several studies all over the world [17–19]. It evaluates 18 activities in 6 categories (self-care, sphincter control, transfers, mobility, communication, and social cognition), which are grouped into two areas, motor and cognitive. Each item is scored on a 1–7 scale regarding the level of assistance required for the individual to perform the particular activity of daily living (1 indicates full assistance and 7 indicates full independence). The total score therefore ranges between 18 and 126 points. FIM scores differentiate between disabilities and levels of severity of impairment, correlate with the time taken for care, and correlate highly with the results of other relevant measures [17]. Reliability and validity of the FIM were proven through several studies [20, 21]. It has been found to have high rates of interrater and test-retest reliability (0.95). 

### 2.2. Participants

In Slovenia, there are about 200,000 persons with disabilities and of those about 10,000 have the most severe types of disability [22]. The target population for our research were the persons with disabilities who were/are treated in the Smart Home IRIS. About 200 persons with different disabilities and of different ages are treated in the Smart Home IRIS each year. Among the persons treated in the study period from April to December 2011, 110 met the inclusion criteria listed below and were thus invited to take part in the study. Adults aged 18 years or more with adequate cognitive capacity who have been referred to the Smart Home IRIS for the first time were included. The age limit was imposed because of the financial and legislative autonomy/independence of the participants and because the FIM is normally only used for adults. Normal cognitive capacity was also required, so a score of at least 25 points on the Mini Mental State Examination (MMSE) [23] was another inclusion criterion. The final sample consisted of the 59 participants who agreed to participate, so a 54% response rate was obtained. The set minimum sample size requirement was met and exceeded, thus compensating for the slight loss of power when analysing data using nonparametric (i.e., rank-based; see Section 2.4) instead of the parametric method (i.e., normal distribution-based) that had been used for sample-size calculation.

### 2.3. Procedure

After receiving the ethical approval for the study, the recruitment of the participants began. Anyone who had already been seen in the Smart Home IRIS and met the inclusion criteria was invited to participate in the study. The recruitment letter with a brief introduction and describing the procedure of research was sent by surface mail to those patients. Those who agreed to take part in the research signed the consent form that was sent together with the recruitment letter and mailed it back in the prepared envelope. The FIM and COPM were completed at the outset of each individual's engagement with the Smart Home IRIS. They were administered in the Smart Home IRIS. This was followed by normal exposure to the smart home. Then the assessments were administered to the individuals again between 6 and 12 months from the initial assessment. Our clinical experience shows that this is an adequate period of time for the individual to realise the advised technical solutions or home modifications. The second set of assessments was conducted at the participants' homes. The recruitment letter, the consent form, and all data collection instruments were in Slovenian, so that they were fully understood by all the participants.

### 2.4. Data Analysis

The collected data were analysed using the PASW Statistics 18.0 software (SPSS Inc., IBM, Somers, NY, USA). Descriptive statistics were calculated for all variables and distributions were depicted graphically. Exact Wilcoxon signed-rank test (EWSRT) was used to test the null hypothesis of no change in the dependent variables between the first and the second assessments. In this way, the first two research hypotheses were addressed. In order to test the third research hypothesis, multiple linear regression was used. Three models were built, one for each dependent variable (FIM total score, COPM performance, and COPM satisfaction individual mean score). In each model, the independent variables were the two factors of interest (i.e., diagnosis type and number of ATs), the score at the first assessment (because it was reasonable to believe that the change in the outcome depended on baseline) and participant's age and gender (which also had to be statistically controlled for). Comprehensive regression diagnostics were performed to assure that the assumptions of the model were met. For illustrative purposes only (because the regression models provided proper inference), bivariate association of the number of assistive technologies with the three modelled outcomes were assessed using Spearman rank-correlation (Rho) and depicted using scatter-plots with linear fit.

## 3. Results

**Figure 2:**
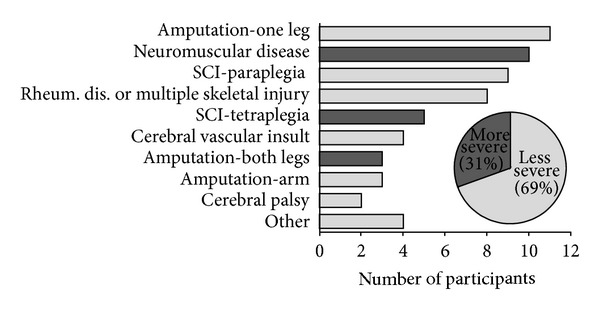
Diagnoses of the participants.

Fifty-nine adult persons with disability participated, 30 men and 29 women. The median age of the participants was 58 years (range 24–81 years). They had different diagnoses; the most frequent diagnosis was amputation of one leg (11 patients, 19%) followed by neuromuscular disease (10 patients, 17%), spinal cord injury (SCI) causing paraplegia (9 patients, 15%), and rheumatic disease or multiple skeletal injury (8 patients, 14%). Other diagnoses included SCI causing tetraplegia, cerebral vascular insult, amputation of one leg or an arm, and cerebral palsy (Figure 2).

For the purpose of further analysis, the diagnoses were divided into two groups: more severe (neuromuscular diseases, spinal cord injury—tetraplegia, and amputation of both legs) and less severe (amputation of one leg, spinal cord injury—paraplegia, multiple skeletal injury and rheumatoid arthritis, cerebral vascular insult, amputation of arm, cerebral palsy, and other). Eighteen participants (31%) belonged to the more severe group and 41 (69%) to the less severe group (Figure 2). In addition to clinical experience, the division was grounded in two established classifications used in the field of rehabilitation that combine medical diagnosis with functional status (FIM score), namely, the Functional Impairment Codes (FIC) and the Australian National Subacute and Nonacute Patient Casemix (AN-SNAP). The key benefit of using the division was that with fewer categories fewer degrees of freedom were spent in the multiple regression models (described further below) applied for testing the third hypothesis, thus making the cases-to-degrees-of-freedom ratio in those models sufficient for a valid analysis.

The number of assistive technologies that the participants used varied from zero (one patient) to five (one patient). The majority of the participants used one (21 patients, 36%) or two assistive technologies (24 patients, 41%). The most frequent assistive technologies were bath and shower seats (used by 31 participants, 53%), grab rails (used by 15 participants, 25%), and adaptations of computer (software and/or hardware; used by 12 participants, 20%).

**Figure 3:**
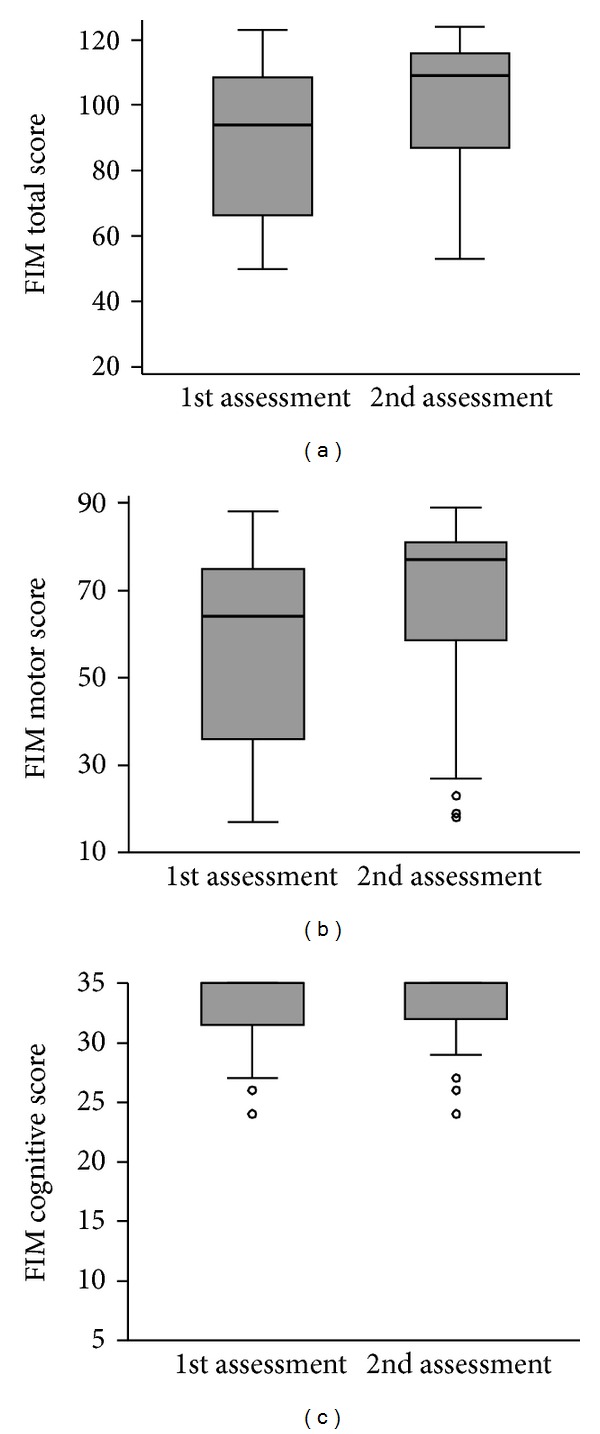
Boxplots of FIM scores at the first and the second assessment (thick line—median; box—interquartile range, IQR; whiskers—values within 1.5 IQR from the 1st and 3rd quartile; circles—outliers).

**Figure 4:**
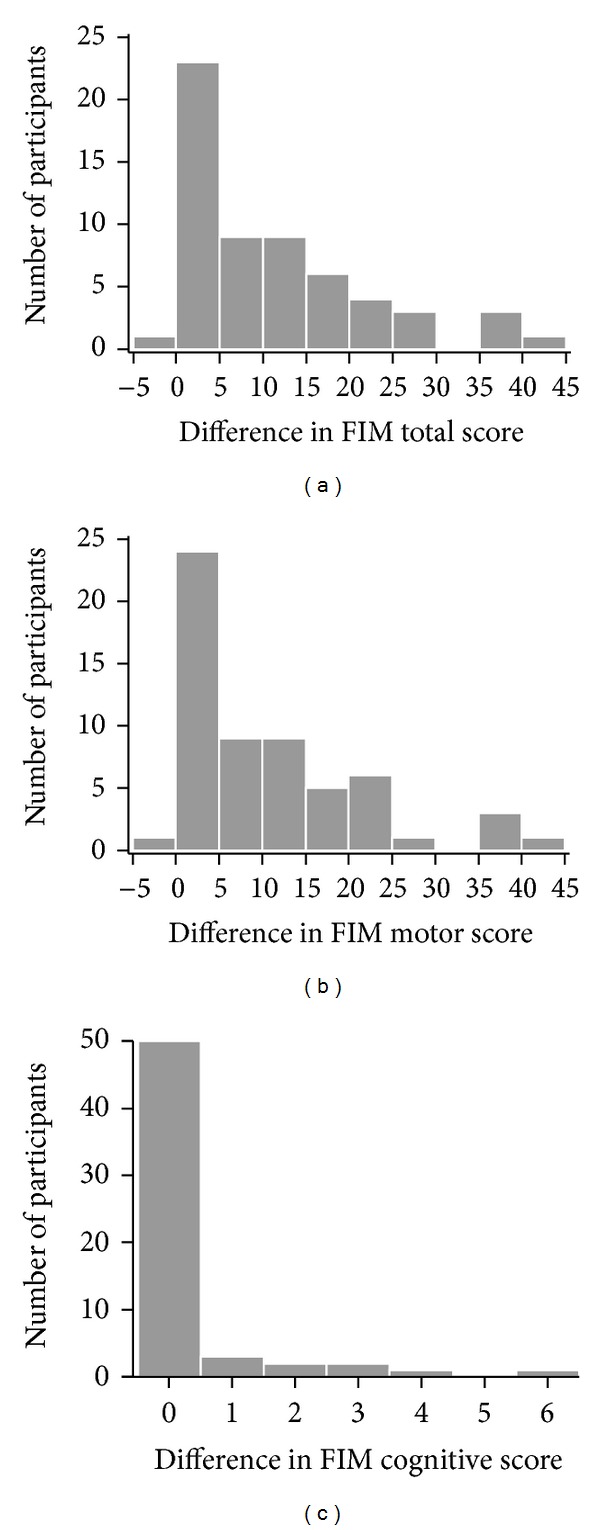
Distribution of differences in FIM scores between the first and the second assessment (histograms for total and motor score, bar chart for cognitive score).

Three FIM scores were analysed—motor sub-score, cognitive subscore and total score. For each of them, the null hypothesis to be statistically tested was that it would not change between the two assessments. All three scores statistically significantly improved (Table 1 and Figure 3). Median score increased by 15 and 13 points for total and motor FIM, respectively (median increase was 7 points for both). Because of the ceiling effect (the maximum possible score was attained by more than half of the patients already at the first assessment), the median cognitive FIM score could not increase (and median increase was zero), but the change in the score was nevertheless statistically significant and the lower quartile as well as the mean score did increase slightly. The distributions of differences in FIM scores between the two assessments (Figure 4) show that motor and total FIM score decreased in only one participant, and that cognitive FIM score did not decrease in any participant.

The problems identified by the participants through COPM were based on occupational performance areas, namely, self-care (personal care, functional mobility, and community management), productivity (work, household management, play, and school), and leisure (recreation, socializing). The number of identified problems varied from one to five; on average, the participants identified three problems. Barriers in home/work environment were identified as a problem by 41 participants (69%); dependence in performing activities of daily living was identified by 37 participants (63%); and limited mobility was identified as a problem by 30 participants (51%). They also identified problems with computer accessibility (17 participants, 29%), communication (8 participants, 14%), and controlling the home environment (6 participants, 8%).

**Figure 5:**
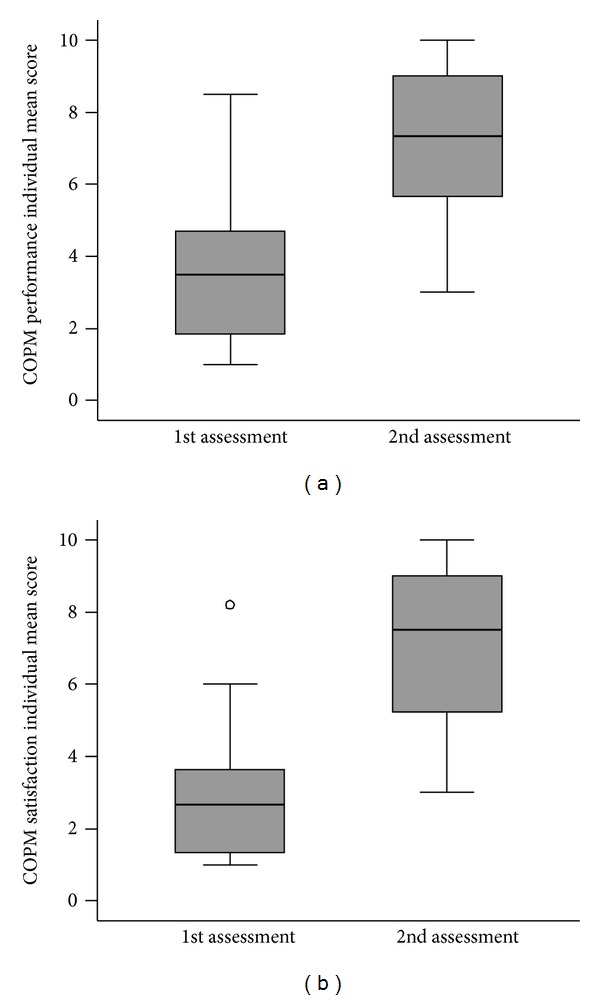
Box plots of COPM individual mean scores at the first and the second assessments (thick line—median; box—IQR: interquartile range, whiskers—values within 1.5 IQR from the 1st and 3rd quartile; circles—outliers).

**Figure 6:**
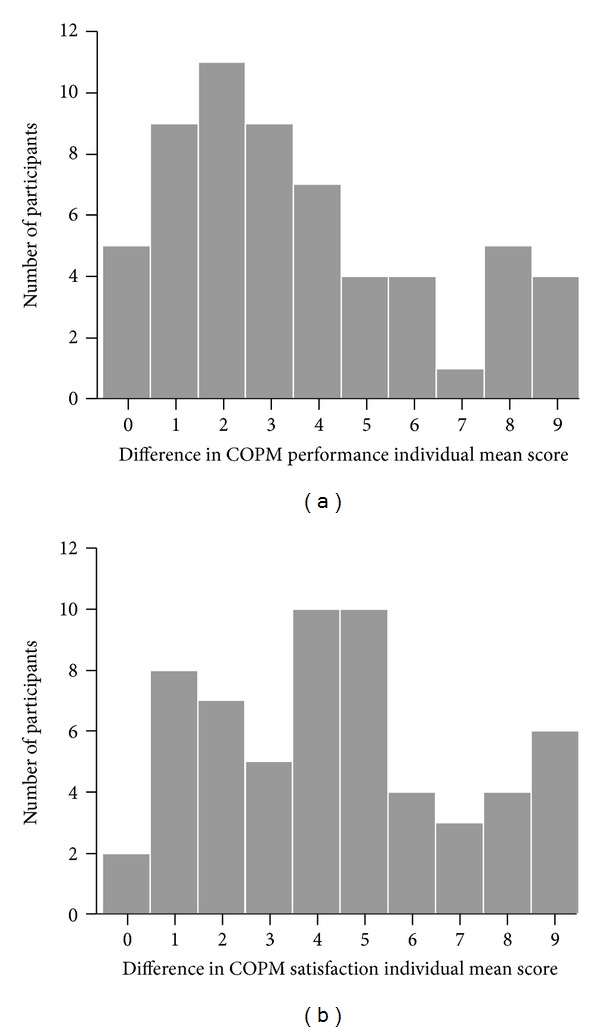
Distribution of differences in COPM individual mean scores between the first and the second assessment (bar charts of rounded values).

Like for the three FIM scores, the null hypothesis to be statistically tested for the two COPM scores was that they would not change between the two assessments. At the second assessment the so-called reflective scoring was used, which means that the participants saw their score of each problem from the first assessment and then they scored the same problem taking into account the previous score. Both performance and satisfaction scores clearly and statistically significantly improved (Table 2 and Figure 5; the median and the mean both increased by about 4; the median increase was 3 for performance and 4 for satisfaction). The distributions of differences in COPM individual mean scores between the two assessments (Figure 6) show that neither performance nor satisfaction individual mean score decreased in any participant.

Multiple regression models for progress in the dependent variables were fitted next. Because of the ceiling effect for the cognitive FIM subscore and because a model for the motor FIM subscore would yield practically identical results, only the total FIM score was modelled as a comprehensive measure of independence in functioning. Complete regression diagnostics were performed, which showed that the data did not substantially violate the assumptions in any of the models. In all three models, VIF values were close to 1 and therefore far below the critical value of 5 (or even 10) that would indicate colinearity (last column in Tables 4, 5, and 6); Durbin-Watson statistic was close to 2, thus indicating no serial correlation (Table 3). Distributions of standardised residuals were not markedly skewed and no standardised residual was below −3 or above 3 (nearly all were between −2 and 2), and scatter-plots of standardised residuals showed no clear indication of heteroscedasticity (all were approximately band-shaped).

All three models were statistically significant (i.e., the null model of predicting the mean of the dependent variable for all cases was rejected at *P* < 0.001; Table 3). The models explained an estimated 33%, 49%, and 36% of population variance for FIM total score, COPM performance individual mean score, and COPM satisfaction individual mean score, respectively (adjusted *R* ^2^ in Table 3). This percentages are quite high given the simplicity of the models (dictated by the limited sample size and available data), which speaks in favour of their validity. On the other hand, it is obvious that a substantial part of variance remains unexplained due to numerous individual and environmental factors that were not assessed and thus not included in the models.

In the model for FIM total score, diagnosis type was a statistically significant predictor (*P* = 0.003; Table 4), while the number of AT was marginally significant (*P* = 0.061; Table 4). The third research hypothesis could therefore be confirmed for FIM total score, whereby the estimated progress for patients in the more severe group was about 8 points less than for those in the less severe group, and for an additional AT the estimated progress was about 2 points larger, both given that all the other parameters in the model were held constant. The statistical significance of the baseline score (*P* = 0.001) is at least partly spurious, that is, artefact, because the difference score and either of its components are correlated by definition, so it should not be over-interpreted and only serves to adjust the patients' progress for the differences in initial functional independence.

The two models for COPM individual mean scores gave essentially equivalent results (Tables 5 and 6). In neither of the models for progress in COPM, diagnosis type was a statistically significant predictor (*P* = 0.147 and *P* = 0.439 for performance and satisfaction, resp.), whereby the estimated regression coefficient was negative (i.e., less progress predicted for the more severe group) as expected in both models. Because the number of ATs was statistically significant in both models (*P* = 0.012 and *P* = 0.022 for performance and satisfaction, resp., with expected progress about 0.7 points larger per additional AT for both outcomes), the third research hypothesis can therefore be at least partly confirmed for COPM scores as well. On a minor note, unlike in the model for FIM score, age was not a statistically significant predictor (*P* = 0.936 and *P* = 0.754), while there was a (nearly) statistically significant gender difference (with higher progress predicted for men) in both COPM models (*P* = 0.034 for performance; *P* = 0.071 for satisfaction). Like the finding regarding age for FIM progress, interpretation of gender difference in COPM progress is difficult and outside the scope of the present research. The same goes for the effect of the baseline score (for the reason outlined in the case of FIM), which was further inflated in case of COPM by the reflective scoring procedure (i.e., the patients seeing their scores at the first assessment during the second assessment), so the largest estimated standardised regression should not be interpreted in terms of relative predictor importance or even causality.

**Figure 7:**
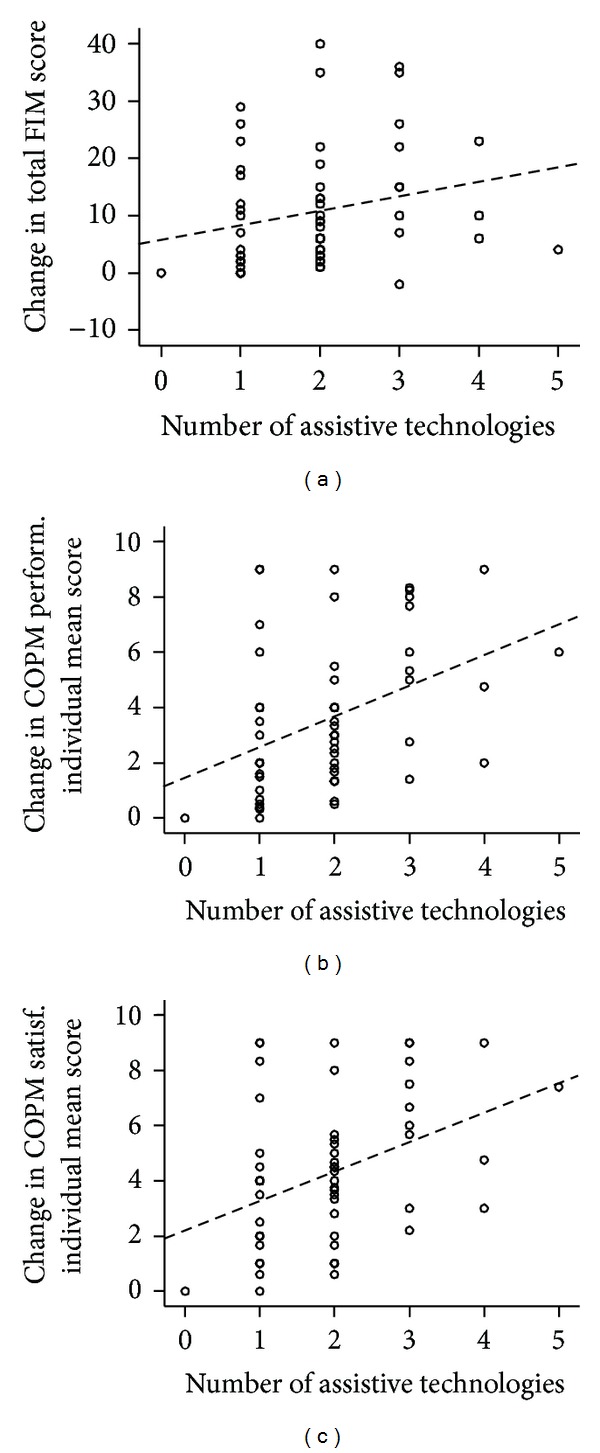
Scatter plots with linear regression lines (dashed; for illustrative purposes only) depicting association of the number of assistive technologies with progress in the outcome measures.

Finally, only for clarification and illustration, bivariate (i.e., unadjusted) analysis of association of the number of ATs with the change scores was performed. The scatter plots with fitted regression lines (Figure 7) and the Spearman correlation coefficients (Rho = 0.328 for change in FIM total score, *P* = 0.011; Rho = 0.424 for change in COPM performance individual mean score, *P* = 0.001; and Rho  =  0.387 for change in COPM satisfaction individual mean score; *P* = 0.002) agree with the finding from the regression models that the more ATs a patient uses, the more progress can be expected from him or her. The association is far from perfect, but it is clear especially for the two COPM scores.

## 4. Discussion

The aim of this study was to evaluate the treatments in the Smart Home IRIS in terms of their effect on occupational performance and functional independence of the treated persons. Three research hypotheses were addressed.

The first hypothesis stated that use of ATs and home modification has impact on increased functional independence for participants who have been treated in Smart Home IRIS. After the second assessment the participants showed a statistically significant improvement in all three FIM scores—total (*P* < 0.001), motor (*P* < 0.001) and cognitive (*P* = 0.004). Though clearly beyond what would could be expected by chance (i.e., due to sampling error), the cognitive FIM could not increase much because of the ceiling effect (maximum possible score was attained by more than half of the patients already at the first assessment). Nevertheless, the increase in median score by 15 and 13 points for the total and the motor FIM, respectively, leaves no doubt that the observed improvement was substantial. In addition, the motor and the total FIM score decreased in only one participant, and the cognitive FIM score did not decrease in any participant. These findings indicate that the participants achieved a higher level of functional independence at the second assessment than at the first assessment. Hence, the first research hypothesis was supported, even though the causal link with the treatment in Smart Home IRIS cannot be firmly established because of the lack of a control group or other means to eliminate (or subtract) the possible effect of other factors.

The second hypothesis stated that the use of ATs and home modification has impact on occupational performance and satisfaction with occupational performance, which was assessed using the COPM. At the second assessment, both the individual mean performance scores and the individual mean satisfaction scores were statistically significantly higher than at the first assessment (*P* < 0.001). The results also showed that neither performance nor satisfaction individual mean score decreased in any participant, which is very important for participants with progressive diseases (such as neuromuscular diseases). These findings are consistent with the findings of the previous studies [24–26]. The second hypothesis was therefore also supported, subject to the same cautionary note regarding causal interpretation as the first hypothesis.

The third hypothesis addressed the differences in progress with regard to the participant's characteristics, focusing on the diagnosis and the number of ATs while controlling for the possible confounding effect of gender, age, and score at the first assessment. Regarding functional independence, the difference with regard to diagnosis type (i.e., the difference between more severe diagnoses, namely, neuromuscular diseases, spinal cord injury causing tetraplegia, and amputation of both legs, and less severe diagnoses) appeared to be more certain (*P* = 0.003) than the positive effect of the number of ATs (*P* = 0.061). The opposite was observed with COPM individual mean scores: the difference with respect to diagnosis type was not statistically significant (*P* = 0.147 and *P* = 0.439 for performance and satisfaction, resp.) though it was in the same direction of more expected progress with less severe diagnoses, whereas the positive effect of the number of ATs was statistically significant in both models (*P* = 0.012 and *P* = 0.022). Nevertheless, the overall picture was the same and agreed with the third research hypothesis.

As already stressed, the association of the baseline score (i.e., at the first assessment) with the progress (i.e., the difference between the second and the first assessment) should not be overinterpreted because of purely mathematical reasons. In addition, the association may not be entirely related with the use of ATs but also with other factors, such as supplementary rehabilitation, concomitant health problems, and natural disease progress. Furthermore, as also already stressed regarding the two COPM scores, the effect of the baseline score status may be overestimated because of the reflective scoring procedure.

There are also other notable limitations to our study. We only considered the number of ATs in the statistical models, thus not taking into account the differences in the impact that a specific AT can have on functional independence. Having more devices does not necessarily lead to increased independence or satisfaction, because the change in independence is based on the match between the AT and the individual's needs, as well as on the degree to which the individual perceives that as leading to more independence. Hence, the distinction between useful ATs and those that are less may be blurred to some extent, which may also be reflected in the less evident positive statistical effect noted for the number of ATs.

It should also be noted that in addition to the number of ATs that a patients possesses and their appropriateness, better occupational performance and higher satisfaction with performance are impacted by the time of application of the ATs prescribed by the occupational therapist or other health care professionals. Appropriate ATs should be provided at the right time, considering the context, activity demands, and client factors (e.g., nature and prognosis of disease/disability) [27]. Late application of ATs leads to less effective usage or abandonment of the prescribed ATs [28]. Another important factor is the way of prescribing ATs, whereby each AT should be recommended and prescribed using a client-centred approach [29]. The present study does not directly assess timeliness of AT provision, though it does underline the need for individual and client-centred approach by occupational therapists and other health professionals who prescribe AT.

As already noted, the treatment in Smart Home IRIS cannot be firmly causally linked with the observed improvements, so a further limitation of our study is that it provides no evidence that factors which were not studied—such as capability of home care staff, specific AT factors (reliability/malfunctions and appropriateness with regard to the consumer's needs), and suitability of home environment—were not important in achieving the observed changes in functional independence, performance, and satisfaction with performance.

Finally, our choice of FIM as the measure of functional independence when using ATs and home modifications is also open to debate. It is not possible to obtain the highest score of 7 (i.e., attain the highest level of independence) on several FIM items from the motor subscale (and hence attain the highest possible motor and total score) if assistive technology is used. Moreover, the FIM is primarily a medical assessment tool not aimed at AT evaluation. Measures such as the Psychosocial Impact of Assistive Devices Scale (PIADS) and Quebec User Evaluation of Satisfaction with Assistive Technology (QUEST) might have been used, which have demonstrated reliability and validity and have been used in evaluating the outcomes of AT interventions [30–32]. However, they have not been adapted for Slovenian language and environment yet, whereas the use of FIM has a relatively long tradition and the years of mandatory assessment of every rehabilitation inpatient at the University Rehabilitation Institute in Ljubljana, accompanied by extensive statistical analyses, vouch for the highest possible level of validity and reliability of the assessment procedure [33, 34].

## 5. Conclusions

The results showed that the use of assistive technologies and home modifications appears to have impact on increased functional independence and better performance and satisfaction. In addition, it was shown that progress differs with respect to the person's diagnosis and the number of assistive technologies he or she uses.

The findings obtained from the present study are important for Slovenian rehabilitation medicine and all health care professionals who work in the field of provision with assistive technologies in Slovenia. We attempted to fill the gap regarding the evidence on effectiveness of a smart home and our results suggest that the persons who use assistive technologies and home modifications may benefit from the treatment in Smart Home IRIS.

As the development of assistive technologies and smart home technologies is spreading, and rehabilitation professionals over the world are becoming aware of the benefits of assistive technologies, continued research in this area is essential. Further high-quality outcome studies, such as randomised controlled trials and longitudinal studies, would be beneficial. It would also be interesting to know whether using assistive technologies and home modifications for a longer period of time (at least for two years) results in long-term improvement.

## Figures and Tables

**Table 1:** Descriptive statistics and results of statistical tests for FIM scores.

Score	Assessment	Mean (SD)	Median (IQR)	*P* (EWSRT)
FIM total (possible range 18–126)	1st	89.6 (21.3)	94 (61–109)	<0.001
	2nd	100.2 (21.0)	109 (84–116)	
	Difference	10.6 (10.5)	7 (2–15)	

FIM motor (possible range 13–91)	1st	56.7 (21.3)	64 (34–75)	<0.001
	2nd	66.9 (21.0)	77 (57–81)	
	Difference	10.2 (10.3)	7 (2–15)	

FIM cognitive (possible range 5–35)	1st	32.9 (2.9)	35 (31–35)	0.004
	2nd	33.3 (2.6)	35 (32–35)	
	Difference	0.4 (1.1)	0 (0-0)	

IQR: interquartile range; EWSRT: exact Wilcoxon signed-rank test; difference = value at 2nd assessment − value at 1st assessment.

**Table 2:** Descriptive statistics and results of statistical tests for COPM individual mean scores.

COPM	Assessment	Mean (SD)	Median (IQR)	*P* (EWSRT)
Performance (possible range 1–10)	1st	3.5 (1.9)	3.5 (1.7–4.8)	<0.001
	2nd	7.1 (2.0)	5.7 (7.3–9.0)	
	Difference	3.6 (2.7)	3.0 (1.5–5.3)	

Satisfaction (possible range 1–10)	1st	2.9 (1.8)	1.3 (2.7–3.7)	<0.001
	2nd	7.2 (2.2)	5.2 (7.5–9.0)	
	Difference	4.3 (2.6)	4.0 (2.0–5.7)	

IQR: interquartile range; EWSRT: exact Wilcoxon signed-rank test; difference = value at 2nd assessment − value at 1st assessment.

**Table 3:** Summary of multiple regression models.

Dependent variable	Adj. *R* ^2^	*P* (ANOVA)	Durbin-Watson
Change in FIM total score	0.325	<0.001	1.913
Change in COPM performance individual mean score	0.489	<0.001	2.432
Change in COPM satisfaction individual mean score	0.360	<0.001	2.688

Adj. *R* ^2^: adjusted coefficient of determination; *P* (ANOVA): statistical significance of the model as a whole; Durbin-Watson statistic: diagnostics of serial correlation.

**Table 4:** Parameter estimates and regression diagnostics of colinearity for multiple linear regression model of change in total FIM score.

Predictor	*b*	*β*	*P*	VIF
Constant	19.646		0.021	

Gender (male versus female)	0.559	0.027	0.816	1.141
Age (years)	0.244	0.352	0.002	1.030
FIM total score at 1st assessment	0.187	0.381	0.001	1.088

Diagnosis type (more versus less severe)	−8.267	−0.366	0.003	1.192
Number of assistive technologies	2.245	0.208	0.061	1.011

*b*: regression coefficient; *β*: standardised regression coefficient; VIF: variance inflation factor.

**Table 5:** Parameter estimates and regression diagnostics of colinearity for multiple linear regression model of change in COPM performance individual mean score.

Predictor	*b*	*β*	*P*	VIF
Constant	4.186		0.006	

Gender (male versus female)	1.174	0.221	0.034	1.168
Age (years)	−0.001	−0.008	0.936	1.031
COPM performance at 1st assessment	−0.757	−0.534	<0.001	1.118

Diagnosis type (more versus less severe)	−0.848	−0.147	0.147	1.126
Number of assistive technologies	0.697	0.252	0.012	1.064

*b*: regression coefficient; *β*: standardised regression coefficient; VIF: variance inflation factor.

**Table 6:** Parameter estimates and regression diagnostics of colinearity for multiple linear regression model of change in COPM satisfaction individual mean score.

Predictor	*b*	*β*	*P*	VIF
Constant	4.236		0.012	

Gender (male versus female)	1.089	0.208	0.071	1.154
Age (years)	−0.006	−0.034	0.754	1.035
COPM satisfaction at 1st assessment	−0.683	−0.454	<0.001	1.140

Diagnosis type (more versus less severe)	−0.498	−0.088	0.439	1.145
Number of assistive technologies	0.696	0.256	0.022	1.076

*b*: regression coefficient; *β*: standardised regression coefficient; VIF: variance inflation factor.
